# Surveillance of health-care associated infections in an intensive care unit at a tertiary care hospital in Central India

**DOI:** 10.3205/dgkh000454

**Published:** 2023-11-29

**Authors:** Ruchita Lohiya, Vijayshri Deotale

**Affiliations:** 1 Dept of Microbiology, Mahatma Gandhi Institute of Medical Sciences, Sewagram, Maharashtra, India

**Keywords:** central line-associated bloodstream infection, CLABSI, catheter-associated urinary tract infections, CAUTI, ventilator-associated pneumonia, VAP

## Abstract

**Introduction:**

Because the risk of health-care associated infections (HAIs) is high in intensive care units, and HAIs are one of the causes of morbidity and mortality and affects the overall quality of health care, the continuous monitoring of HAIs in intensive care patients is essential.

**Aim and objectives:**

This descriptive cross-sectional study was carried out over a period of five years in a tertiary-care teaching hospital. The aim of the study was to investigate the main and specific types of health-care associated Infections and determine the microbiological profile and antimicrobial susceptibility rates of isolates in patients with HAI.

**Methods:**

: The active surveillance method was used to detect HAIs in patients who spent over 48 hr in a targeted ICU. Patients with blood stream infections (BSI), central line-associated bloodstream infection (CLABSI), catheter-associated urinary tract infections (CAUTI) and ventilator-associated events (VAE) were included in the study. HAI were diagnosed based on the Centre for Disease Control (CDC)’s National Healthcare Safety Network (NHSN) updated definitions of HAIs.

**Results:**

A total of 121,051 patient days, including 7,989 central line days, 64,557 urinary catheter days, and 18,443 ventilator days, were recorded in the study population and 832 HAIs were diagnosed (incidence rate 6.9%). The overall rates of BSI, CLABSI, CAUTI and possible ventilator-associated pneumonia (p-VAP) were 3.7, 10.6, 2.1 and 13.4/1,000 device days, respectively. The most common organism isolated from BSI was *Acinetobacter baumanii* (n=322, 29%), followed by *Klebsiella pneumoniae* 225 (n=225, 20.3%). 79.8% of *Acinetobacter baumanii* strains were resistant to imipenem, 77.1% to ciprofloxacin and 76.4% to ampicillin. The most common organisms isolated from CAUTI were non-albicans *Candida *species (n=38, 18%), followed by *E. coli* and *Citrobacter *spp*.* (each n=33, each 15.7%).

**Conclusions:**

A trend of increasing resistance of *Acinetobacter baumannii* to carbapenems was observed. Risk factor analysis showed invasive procedures during sepsis and organophosphorous poisoning as significant factors.

## Zusammenfassung

**Einleitung:**

Weil die Gefährdung durch Health-care assoziierte Infektionen (HAI) in Intensivtherapieeinheiten besonders hoch ist, HAI eine wesentliche Ursache für Morbidität und Letalität sind und die Gesamtqualität der Gesundheitsversorgung durch HAI beeinträchtigt wird, ist ein kontinuierliches Monitoring von HAI bei Intensivtherapiepatienten unerlässlich.

**Zielsetzung:**

Die deskriptive Querschnittsstudie wurde über einen Zeitraum von fünf Jahren in einem Lehrkrankenhaus der Tertiärversorgung durchgeführt. Ziel war es, die wichtigsten und spezifischen Arten von HAI zu untersuchen sowie das mikrobiologische Profil und die Antibiotikaempfindlichkeit der Isolate zu bestimmen.

**Methode:**

Es wurde die Methode der aktiven Surveillance eingesetzt, um HAI bei Patienten zu erkennen, die mehr als 48 h auf den Intensivstationen untergebracht waren. In die Studie wurden Patienten mit Blutstrominfektionen (BSI), ZVK-assoziierten Infektionen (CLABSI), Katheter-assoziierten Harnweginfektionen (CAUTI) und Beatmungsassoziierte Pneumonien (VAP) aufgenommen. Die HAI wurden auf der Grundlage der aktuellen Definitionen für HAI des National Healthcare Safety Network (NHSN) des Centers for Disease Control (CDC) diagnostiziert.

**Ergebnisse:**

Es wurden 121.051 Patiententage, 7.989 Zentralvenenkathetertage, 64.557 Harnkathetertage, 18.443 Beatmungstage erfasst und 832 HAI diagnostiziert (Inzidenzrate 6,9%). Die Gesamtraten von BSI, CLABSI, CAUTI und möglicher VAP betrugen 3,7, 10,6, 2,1 bzw. 13,4/1.000 Devicetage. Die am häufigsten bei Blutstrominfektionen isolierte Species war *Acinetobacter baumanii* (n=322, 29 %), gefolgt von *Klebsiella pneumoniae* (n=225, 20,3%). 79,8% der *Acinetobacter baumani*i Stämme waren gegen Imipenem, 77,1% gegen Ciprofloxacin und 76,4% gegen Ampicillin resistent. Die häufigsten Mikroorganismen, die bei CAUTI isoliert wurden, waren non-albicans *Candida-*Spezies (n=38; 18%), gefolgt von *E. coli* und *Citrobacter *spp*.* (je n=33; je15,7%).

**Schlussfolgerung:**

Es wurde ein Trend zur zunehmenden Resistenz von *Acinetobacter baumannii* gegen Carbapeneme beobachtet. Die Analyse der Risikofaktoren ergab, dass invasive Eingriffe während Sepsis und phosphororganische Vergiftungen einen wichtigen Risikofaktor darstellen.

## Introduction

Healthcare-associated infections (HAI) are one of the causes of morbidity and mortality in healthcare settings. Patients who are admitted and treated in intensive care units (ICU) are severely ill, with decreased immunity and many co-morbidities [1]. In these units, the possibility of infecting a patient is higher than in other units [2], [3], [4]. In the ICU environment, medical procedures applied to treat the patient and the patient’s general health are responsible for this increase in HAI in ICUs. ICUs treat severely ill patients whose underlying disease and coexisting diseases may contribute to the development of HAI [5]. Continuous monitoring of ICU patients is essential, as most device application takes place on ICU patients, such as central vascular lines (CVC), intubation tubes, and urethral catheters. In a tertiary care hospital, ICU admission is often preceded by hospitalization in other hospital, during which there are often problems with the treatment of such patients. During the patient’s stay in hospital (regardless of the ward in which they are hospitalized), the physiological bacterial microbiota is replaced with the hospital’s microbiota. In addition, long-term antibiotic treatment leads to selection for less sensitive microorganisms in the patient’s body, which may facilitate the body being colonized by pathogenic microorganisms with proven resistance to antibiotics [6]. Therefore, it is extremely important to undertake prophylactic measures that will at least protect patients from the negative health effects of microorganisms from the hospital environment; this is especially true for patients in ICUs, where the invasiveness of treatments and reduced immunity of the patient predispose them to becoming colonized by such bacteria and acquiring infections [7].

Apart from healthcare-associated factors contributing to HAI, environmental factors and patient-related factors are also important. Environmental factors include contaminated air-conditioning systems and overcrowding due to the improper infrastructure design of the facility, lack of health-care workers (HCW), and lack of effective intervention programs designed to reduce HAI. Patient-related factors comprise severity of underlying illness, use of immunosuppressive agents, and prolonged hospital stays and prolonged antibiotic administration.

According to the WHO, the global HAI burden is 7–12% [8], [9]. In India, these figures are alarming, with incidence rates varying from 11–83% for different kinds of HAI [10]. Frequently prevalent infections in the hospitals include central line-associated bloodstream infections (CLABSI), catheter-associated urinary tract infections (CAUTI), surgical site infections (SSI) and ventilator-associated pneumonia (VAP). There are many ways in which infection control nurses (ICN) can play a part in surveillance and help to monitor these device-associated HAI (DA-HAI) rates. In order to do this effectively, it is vital that they receive appropriate support and training from infection control personnel and existing surveillance and auditing staff.

During hospitalization, the patient is exposed to nosocomial pathogens from different sources, such as the environment, HCW and even cross infection. These infections need to be prevented by using various prophylactic measures, e.g., implementing an infection control program, vigilance towards increased microbial clustering, antimicrobial resistance, and adopting bundled care policies.

## Aim and objectives

Although the burden of HAI is higher in our developing country, active surveillance of this nature is not being conducted in any large tertiary care rural hospital in central India. With this background, this study was conducted to assess the HAI burden in our tertiary care hospital by performing active surveillance with special attention given to the ICU and the device utilization ratio (DUR) using CDC’s NHSN surveillance criteria [11]. An additional aim was to identify nosocomial pathogens associated with DA-HAI.

## Methods

### Study design

Descriptive prospective observational cross-sectional study.

### Site of study

Tertiary care hospital of central India.

### Setting

The active surveillance method was used to detect HAIs in patients who spent over 48 hrs in ICUs, i.e., medical ICU (MICU), surgical ICU (SICU), pediatric ICU (PICU), neonatal ICU (NICU), trauma ICU, neuro ICU, maternity ICU and the cardiac catheterization lab (Cath lab ICU).

### Implementation

For conducting active surveillance in selected ICUs, our team of ICNs was deputed. Before going on rounds, the team marked the patient samples having culture positive of designated ICUs. With the patients’ details from the laboratory, ICNs visited the bedside of the patient. All the details of the patient as per the criteria enlisted for BSI, CLABSI, CAUTI and VAP were collected from the case papers of patients. Using the checklist (Attachment 1, Attachment 2, Attachment 3, Attachment 4, Attachment 5), all the necessary data was recorded. All the forms were analyzed to determine the incidence of BSI, CLABSI, CAUTI and p-VAP.

After approval from the Institutional Ethics Committee (Approval Letter Number: IEC/249/2016), this prospective observational study was conducted in all designated ICUs of the tertiary care hospital in Central India from January 2018–December 2022. This tertiary care hospital comprises 960 beds and eight ICUs. 

### Study population

A total of 27,657patients were admitted to the ICUs for more than 48 hrs, for a total of 121,051 patient days. Patients were followed up to their discharge from ICU or death. The information of each patient regarding their clinical details, antibiotic prescription, operative procedures if any, and date and site of device insertion was noted. Check lists care bundles were noted in the data sheet; in addition to clinical and hospital monitoring details, patients’ demographic information (i.e., gender, age), admission and discharge dates, comorbidity, device use and date of insertion, isolated pathogen and its susceptibility pattern, and infection sites. For estimating DA-HAI, incidence density rates were calculated as per the formula given: p-VAP events were divided by the number of total ventilator days multiplied by 1,000. For CLABSI, the confirmed CLABSI events were divided by total CVC days multiplied by 1,000, and for CAUTI, the confirmed CAUTI events were divided by total urine catheter days multiplied by 1,000. Device utilization days were calculated as total number of device days divided by the total number of patient days [11]. 

According to the approved CDC criteria,

1. Device days are the total number of days of exposure to each device (endotracheal tube, central venous catheter or urinary catheter) for all the patients during the selected time period.
2. Patient days are the total number of days patients are in the ICU during the selected time period [11].

### Sampling and laboratory testing

In suspected cases of BSI, especially in CLABSI, the CVC was aseptically removed and the distal 4 cm of the catheter was separated and cultured. For CAUTI, urine samples were collected aseptically by aspirating the sample from the urine sample port. Quantitative culture for aerobic bacteria was performed using samples of respiratory tract secretions to detect p-VAP [12], [13]. Lower respiratory tract secretions were collected using tracheal aspiration and/or bronchoalveolar lavage whenever applicable. All the isolates were identified using the conventional method [14], and susceptibility tests were performed in all cases [15].

### Data analysis

The data were entered into an excel sheet daily and analyzed at the end of every month to generate HAI rates and DUR. Cumulative HAI and DUR were determined for all the ICUs of the hospital over a period of five years. 

## Results

### Incidence rate of HAIs

During the study period, 27,657 patients were admitted to ICUs, out of which 832 cases of HAI were diagnosed. The HAI incidence rate in the studied population was 6.9%. In the group of 832 HAI cases, 538 (64.6%) were males and 294 (35.4%) were females. The mean age of the patients hospitalized in MICU, SICU, PICU and NICU was 45 yrs, 50 yrs, 3 yrs and 9 days, respectively. The mean ICU stay was 15.5 days. The mortality was amongst 448 (53.8%). Sepsis, ARDS, and poisoning accounted for the majority of the ICU admission diagnoses (39.7%, 17.1%, 14%, respectively; Table 1 (Tab. 1) and Table 2 (Tab. 2)).

**Figure 1:**
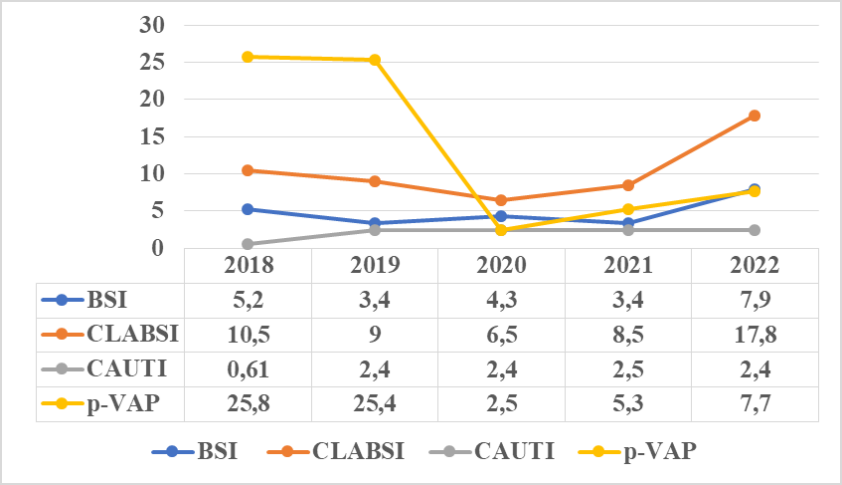
Incidence rates of DA-HAI over the 5-year study period

The mean incidence rates for all ICUs were 3.7 for BSI per 1,000 patient days, 10.6 per 1,000 central line days for CLABSI, 13.4 per 1,000 ventilator days for p-VAP and 2.1 per 1,000 catheter days for CAUTI (Table 3 (Tab. 3), Table 4 (Tab. 4), Table 5 (Tab. 5)). The device application days were 7,989 for the central line catheter, 18,443 for the ventilator and 64,551 days for the urinary catheter, with a device utilization ratio (DUR) of 0.065, 0.15 and 0.53, respectively (Table 3 (Tab. 3), Table 4 (Tab. 4), Table 5 (Tab. 5)). The incidence rate showed a sharp decline from 2019 to 2020 and a gradual increase from 2020 to 2022 (Figure 1 (Fig. 1)). 

### Microbial etiology of HAIs

**Figure 2:**
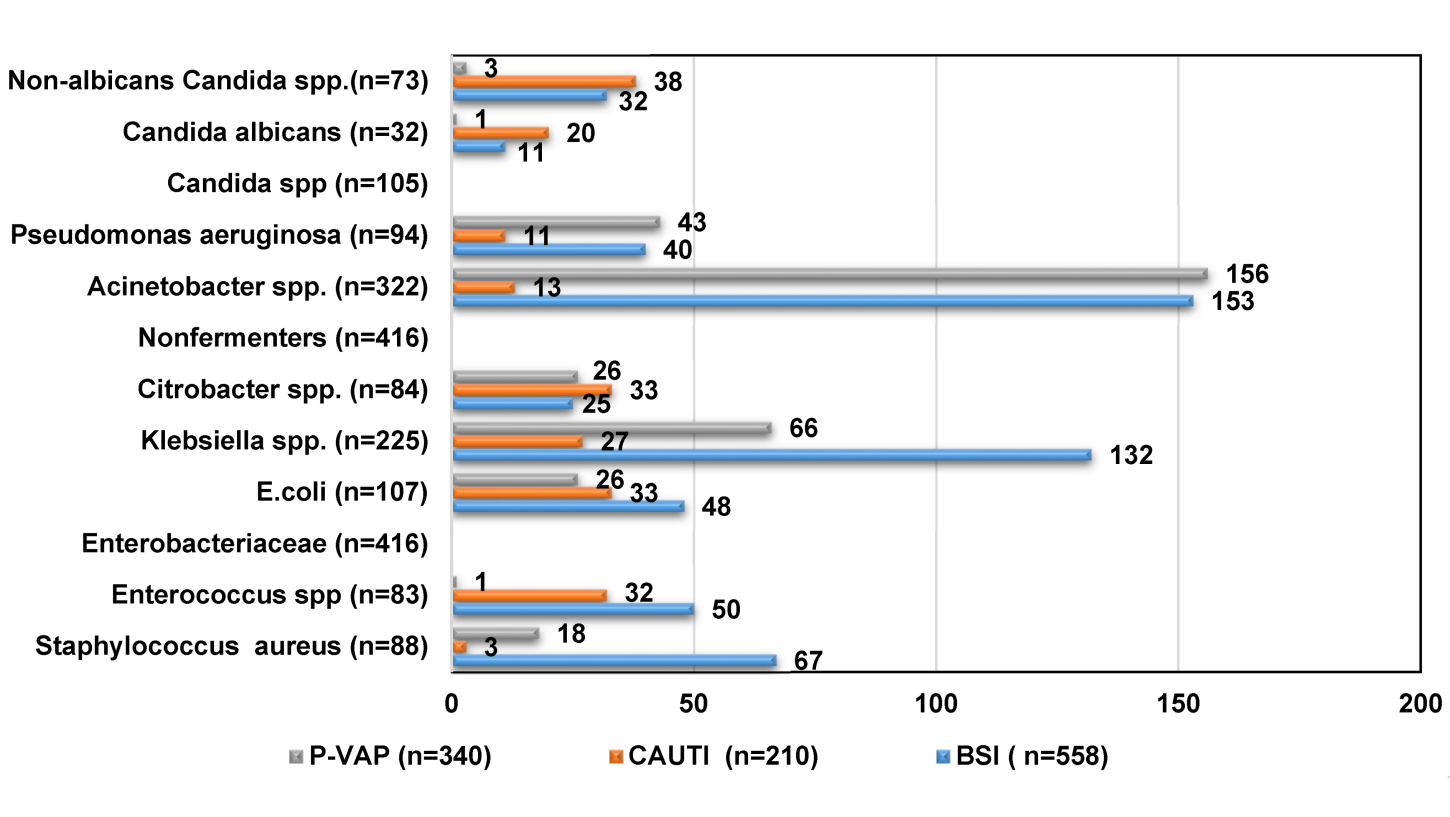
Distribution of HAI pathogens in the ICUs (n=1,108)

Out of 1,108 pathogens, the most frequently isolated were Gram-negatives (n=225, 54%), thereof *E. coli* n=107 (5.7%), *Citrobacter* and *Enterobacter* spp. each n=84, 20.2%). Gram-negative non-fermenting bacteria accounted for n=416, which include *Acinetobacter spp.* (n=322, 77.4%) and *Pseudomonas spp.* (n=94, 22.6%). Among 171 Gram-positive pathogens,* Staphylococcus aureus* accounts for n=88 (51.5%). of which 31% were MRSA. The rest were *Enterococcus *spp. (n=83, 48.5%). Among the fungal pathogens, non-albicans *Candida* spp. was the most frequently isolated with 69.5% (n=73), thereof *C. utilis* (50%), *C. tropicalis* (22.7%), *C. pelliculosa* (13.6%), *C. glabrata* (6.8%), *C. ciferri* (4.5%) and *C. rugosa* (2.3%). The proportion of *C. albicans* among fungi was 30.1% (n=32) (Figure 2 (Fig. 2)).

### Resistance

**Figure 3:**
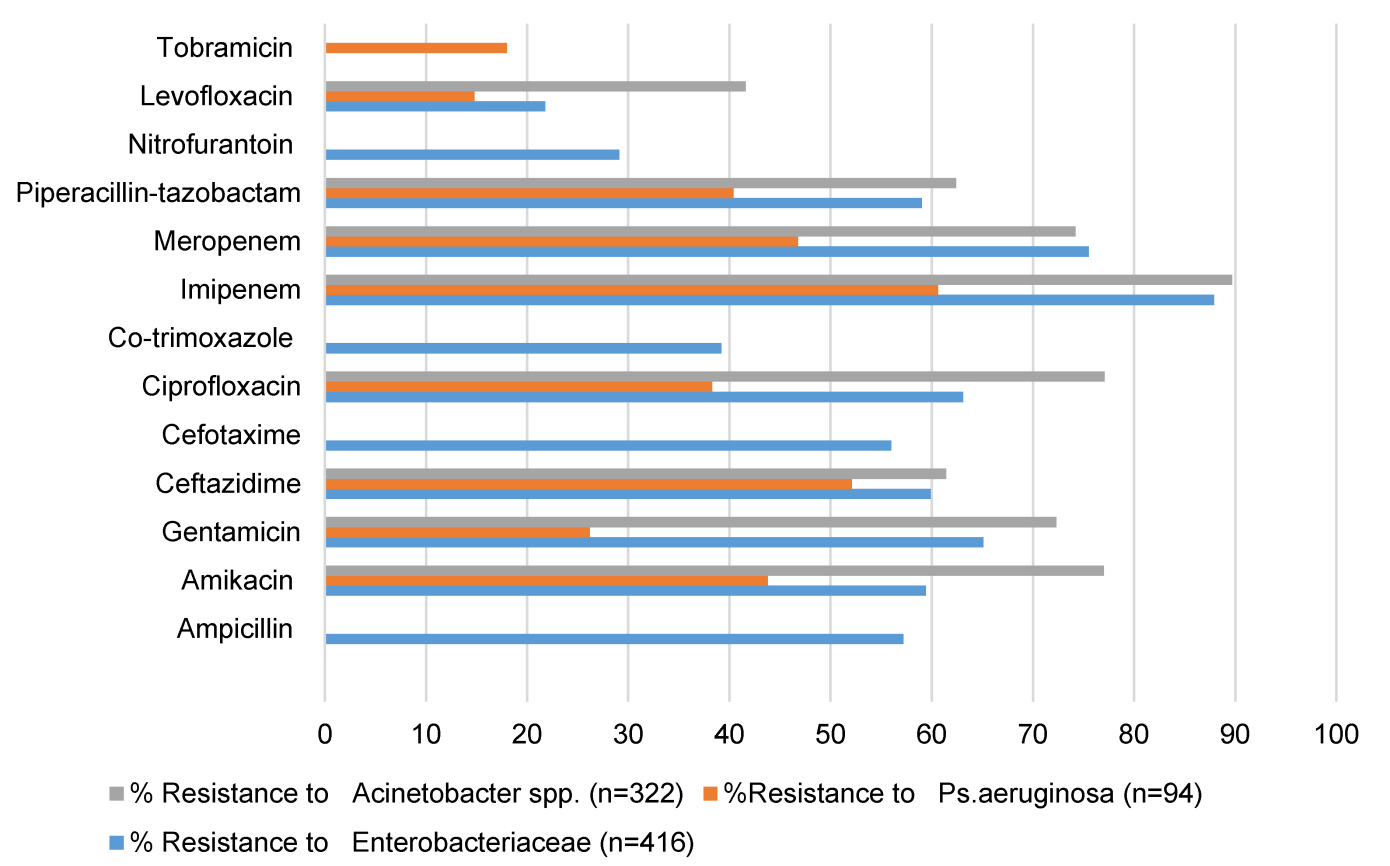
Antimicrobial resistance in Gram-negative organisms isolated from HAI

**Figure 4:**
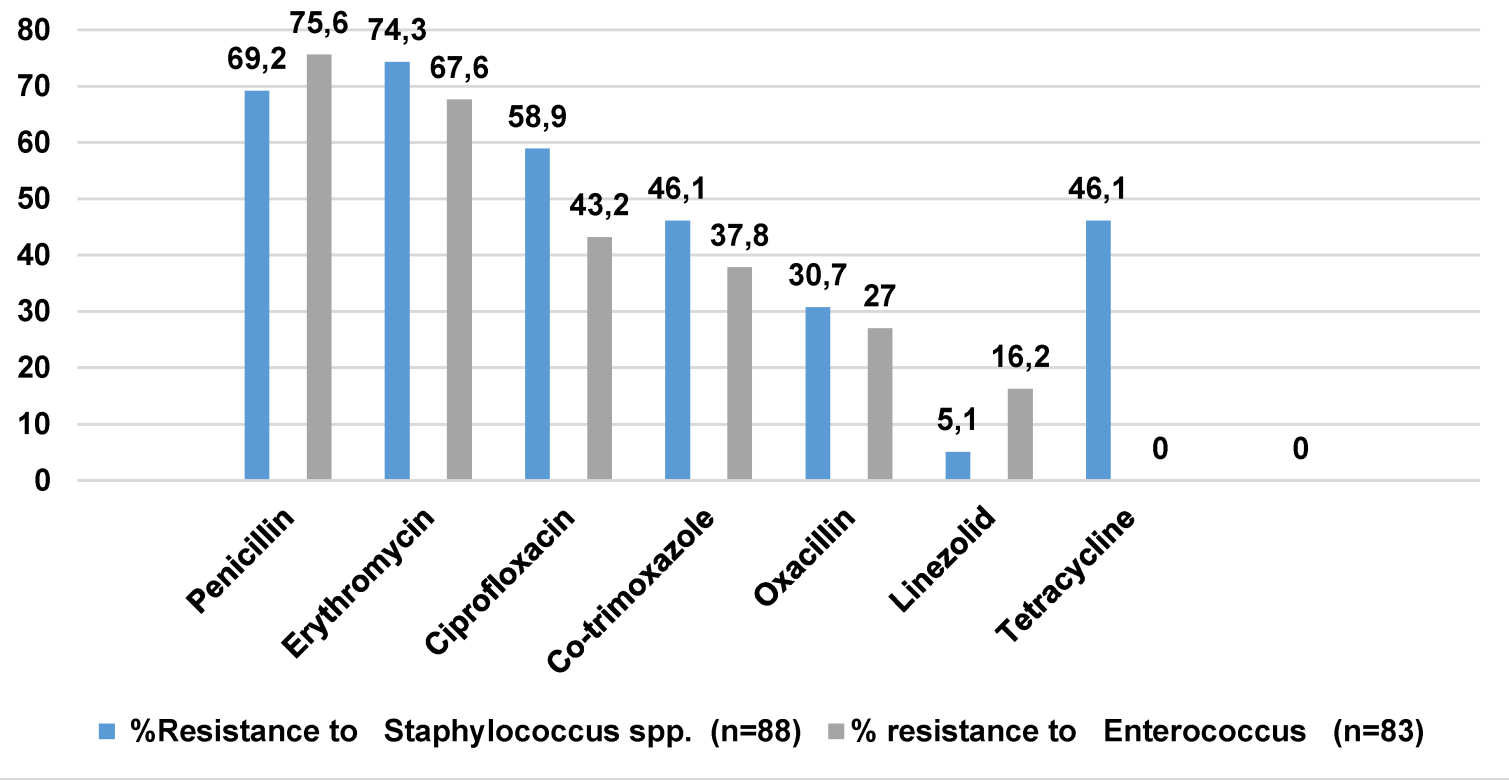
Antimicrobial resistance in Gram-positive organisms isolated from HAI

The bar diagrams presented in Figure 3 (Fig. 3) and Figure 4 (Fig. 4) illustrate the patterns of antimicrobial resistance in Gram-negative and Gram-positive organisms isolated from HAIs. The figures provide valuable insights into the current state of antimicrobial resistance, which is a critical concern in healthcare settings. Figure 3 (Fig. 3) depicts the antimicrobial resistance trends in Gram-negative organisms. The data suggest a disquieting level of resistance across multiple classes of antimicrobials. This is indicative of the growing challenge in treating infections caused by Gram-negative bacteria, as the effectiveness of various antibiotics is compromised. In contrast, Figure 4 (Fig. 4) focuses on antimicrobial resistance in Gram-positive organisms. While resistance is still evident, it might be relatively less pronounced in certain categories of antibiotics. This could be attributed to variations in the mechanisms of resistance and the prevalence of specific strains within Gram-positive bacteria. 

## Discussion

A major problem in hospitalized patients is HAI. This may be due to prolonged hospital stays due to serious ailments or due to invasive procedures, such as mechanical support for a compromised organ system. Therefore, monitoring of HAIs is important in ICUs for improving the outcome of patients. 

This is the largest single center study of HAI from ICUs from a rural tertiary care hospital in Central India. The present study shows the overall incidence of HAI to be 4.62%, which – among the general HAI rate in Indian ICUs – agrees best with International Standards, i.e. 4.4% [16]. Our study reports HAI rates lower than those in other studies, e.g., like Shalini et al. [16] (27.4%) and Singh et al. [17] (17.6%).

The demographic parameters in the present study revealed that the number of males admitted to the ICU was almost double that of females, and the mean age of patients in adult category admitted to MICU and SICU was 45 and 47 yrs, respectively. This finding is concordant with studies conducted by Moolchandani et al. [18], Anand et al. [19] and Patel et al. [20]. The mean ICU stay was 15.5 days. The combined CLABSI rate for all targeted ICUs was found to be 9.28/1,000 patient days and the central line utilization ratio was estimated to be 0.06. Although there are very limited studies on CLABSI from Indian institutions, the incidence of CLABSI varied from 0.2% to 27%, with a rate of 0.5 to 47/1,000 patient days [10]. Table 6 (Tab. 6) compares the rates reported here with those of INICC India [21], NHSN [22] and other studies from India [23]. Our rates correlated well with INICC India, but were significantly higher than NHSN, especially that of p-VAP, due to long-term mechanical ventilation used for patients. The long durations of mechanical ventilation at our hospital is probably related to shortage of adequate nursing staff, which may have adversely affected the quality of care given to the patients. Also, the health-seeking behavior of our patients is different compared with that in developed countries. Patients seek medical help only when it is absolutely inevitable. By the time patient is referred to the tertiary-care centre, her/his underlying condition is exacerbated and may be irreversible. An overall correlation was found between DU ratio and DAI rates with the INICC India survey. In our hospital, there was no significant change in incidence rates of HAI over a 3-year period. The mortality rate was 46.8% in our study. These findings are similar to the crude mortality rate in the INICC survey India, which ranged from 35.2% to 44.9% [10] .

In our study, the device utilization ratio was lower, similar to those of other studies from China, Malaysia and Iran [24], [25], [26]. The utilization ratio for central lines, ventilators and urinary catheters was 0.62, 0.47, and 0.84, respectively. 

All HAIs need to be prevented by using various prophylactic measures, such as practicing an infection control program, vigilance towards increased microbial clustering and their antimicrobial resistance, and by adopting care-bundle policies. These steps will help to promote efficient measures. The infection surveillance and risk factors analysis are important prerequisites for the prevention and treatment of HAIs. Apart from healthcare-associated factors contributing to HAI, environmental factors and patient-related factors (e.g., any comorbidities or an immunocompromised state) are also important. Environmental factors include contaminated air conditioners, overcrowding of patients due to sudden increased inflow of patients, a smaller number of healthcare workers and lack of effective intervention programs to reduce HAI. 

Table 6 (Tab. 6) provides a comparative overview of mean HAI rates per 1,000 patient days across different studies, focusing on CLABSI, CAUTI and VAP. The presented data reveal variations in HAI rates across different healthcare institutions and regions, highlighting the importance of understanding and addressing these infections within diverse contexts. The present study, spanning the years 2018 to 2022, reported mean HAI rates of 10.6 for CLABSI, 2.1 for CAUTI, and 13.4 for p-VAP. Notably, this study shows a substantial reduction in CAUTI rates and a slightly elevated CLABSI rate compared to the All India Institute of Medical Sciences (AIIMS) study, indicating potential successes in CAUTI prevention and areas needing improvement in CLABSI management. This table presents a comprehensive overview of mean HAI rates across different healthcare settings and regions. Variations in rates could arise from differences in infection control practices, patient populations, healthcare infrastructure, and data collection methods. These findings underscore the need for continuous surveillance, targeted interventions, and knowledge sharing to mitigate HAIs effectively and improve patient outcomes across diverse healthcare environments. 

## Conclusion

Specialized procedures are used in ICUs for treatment and diagnosis, involving invasive monitoring and mechanical support of insufficient organ systems, which increase the risk of HAI. Therefore, monitoring of HAI in ICUs is important for improving the outcome of treatment. In our rural hospital, we have established infection prevention measures by conducting prior training among HCW, holding workshops for them, and performing surveillance of DA-HAI. Besides these measures, monitoring of Central Sterile Supply Department (CSSD) is the backbone of decreasing HAI.

The rising resistance to antimicrobials highlights the need for judicious use of antibiotics and the development of novel treatment strategies to combat these infections effectively. This study underscores the urgent need for comprehensive strategies to address antimicrobial resistance in healthcare settings. Effective infection control measures, antibiotic stewardship programs, and continued research into new antimicrobial agents are essential to tackle the growing threat posed by multidrug-resistant organisms. Additionally, these figures emphasize the importance of ongoing surveillance and data collection to monitor the evolving landscape of antimicrobial resistance and inform clinical decision-making.

## Notes

### Competing interests

The authors declare that they have no competing interests.

### Acknowledgements

This work was part of a multicenter project titled “Capacity Building and Strengthening of Hospital Infection Control to Detect and Prevent Antimicrobial Resistance in India” by All India Institute of Medical Sciences (AIIMS) New Delhi, supported by the U.S. Centers for Disease Control and Prevention cooperative agreement NU2GGH001869-01-00 – 2015–2021. The project was technically coordinated by the Indian Council of Medical Research (ICMR), New Delhi. We thank Paul Malpiedi, Daniel Van der Ende, and Siromany Valan (U.S. Centers for Disease Control and Prevention) for the technical support. We also acknowledge the support of Dr. Purva Mathur, AIIMS New Delhi and Dr. Kamini Walia and their team for this work.

### ORCID

- Ruchita Lohiya: 0000-0003-1766-4682
- Vijayshri Deotale: 0000-0002-9347-9836>

## Supplementary Material

Audit form

Daily Round Checklist (ICN)

ICU Checklist

Checklist for Urinary Catheter

Checklist for Central Line

## Figures and Tables

**Table 1:**
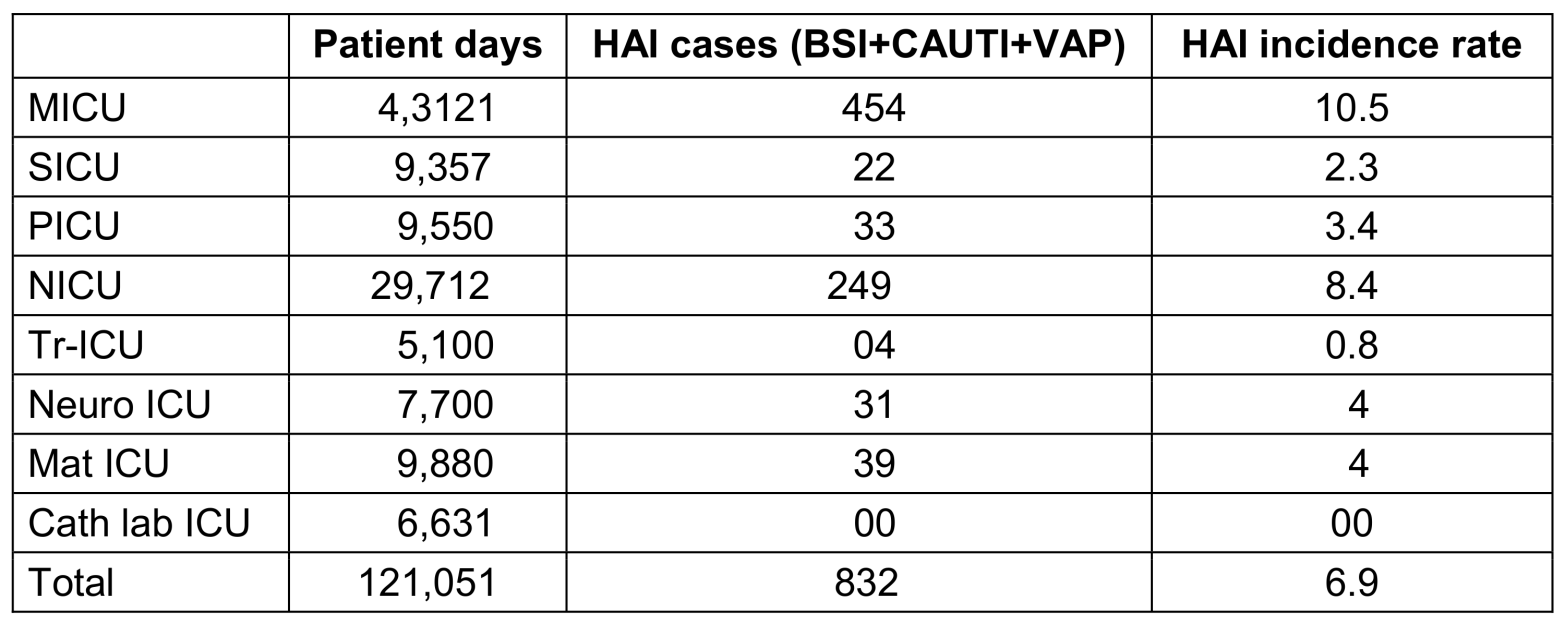
Incidence rate of HAI cases enrolled in the study

**Table 2:**
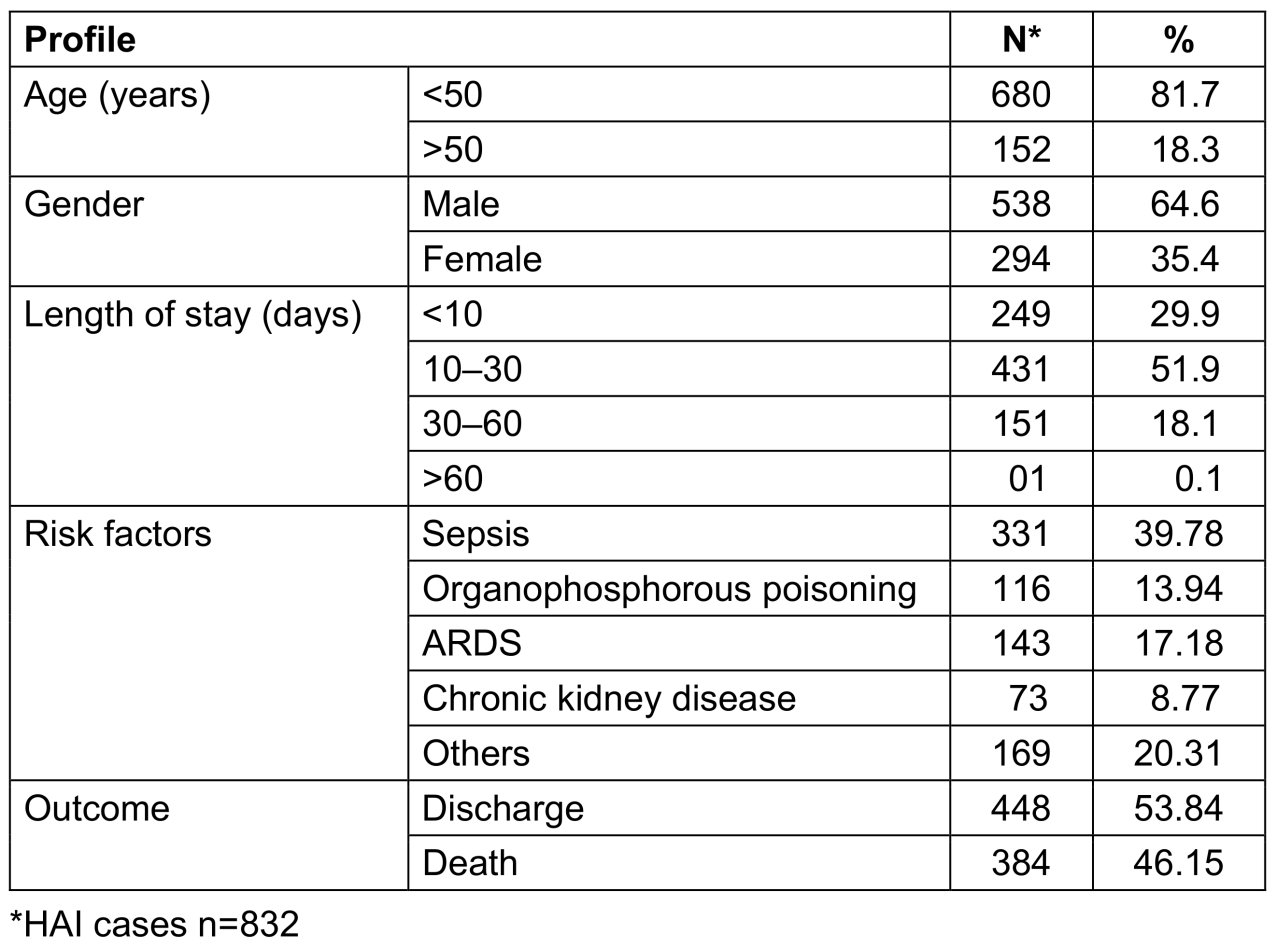
Demographic profile of HAI cases enrolled in the study

**Table 3:**
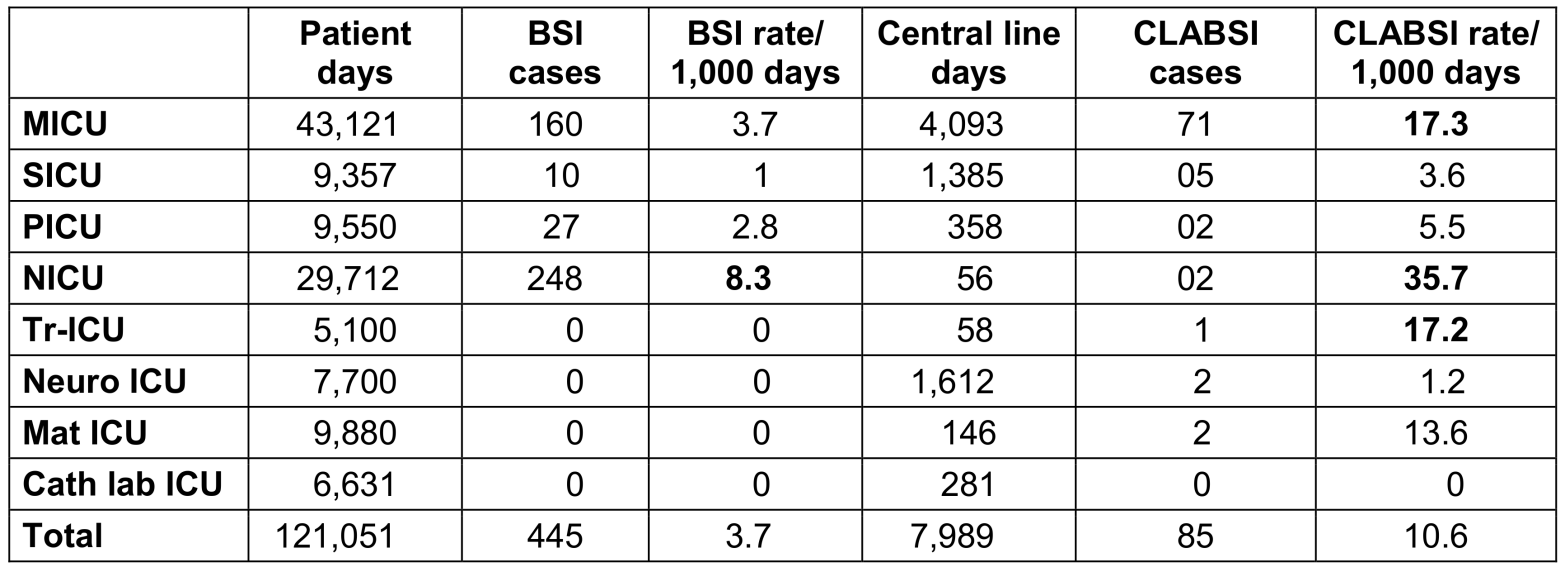
Distribution of BSI and CLABSI in the ICUs

**Table 4:**
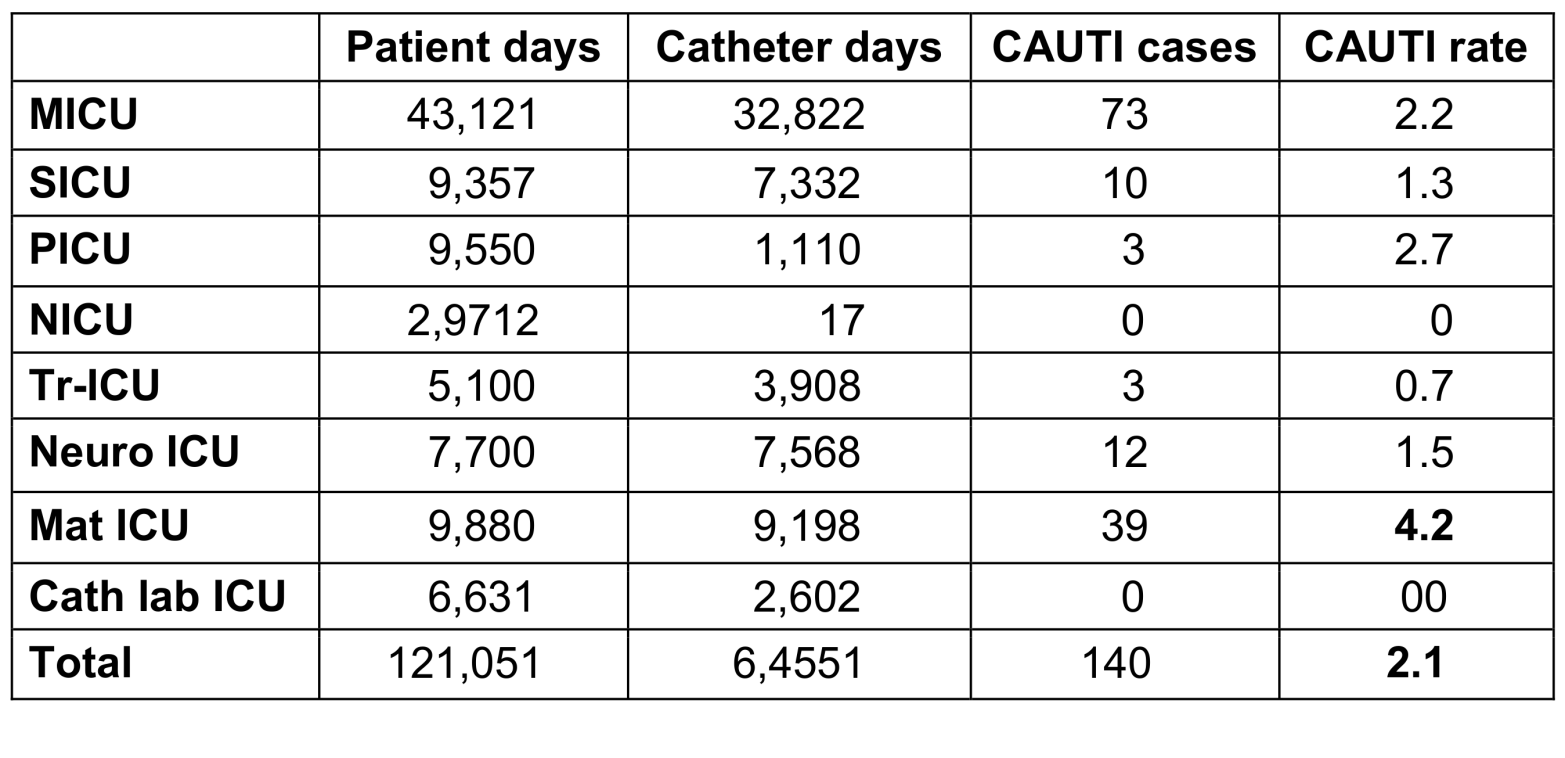
Distribution of CAUTI in the ICUs

**Table 5:**
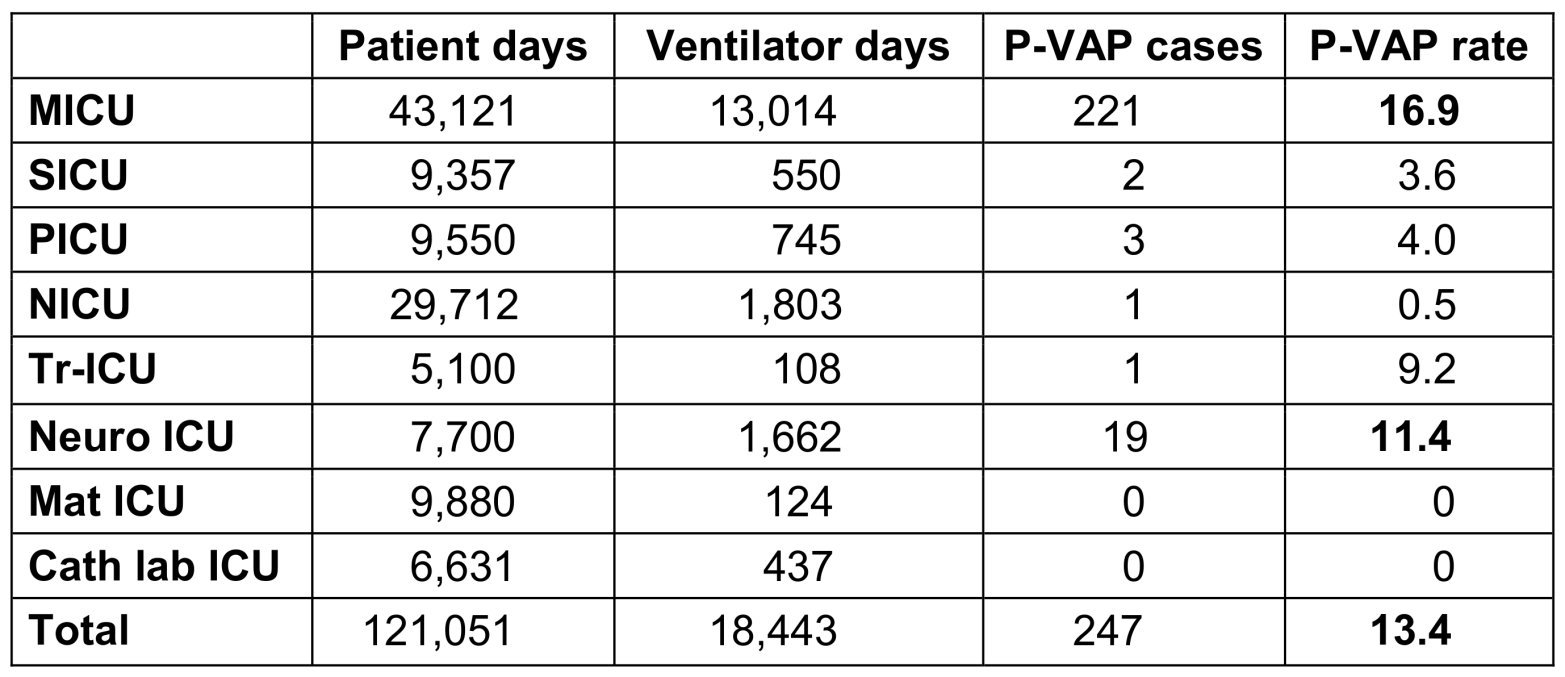
Distribution of p-VAP in the ICUs

**Table 6:**
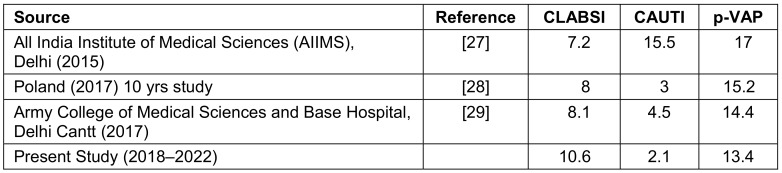
Mean HAI rates/1000 patient days in current and other studies

## References

[1] Prin M, Li G (2016). Complications and in-hospital mortality in trauma patients treated in intensive care units in the United States, 2013. Inj Epidemiol.

[2] Allegranzi B, Bagheri Nejdar S, Castillejos GG (2011). Report on the burden of endemic health care-associated infection worldwide. A systematic review of the literature.

[3] Bammigatti C, Doradla S, Belgode HN, Kumar H, Swaminathan RP (2017). Healthcare Associated Infections in a Resource Limited Setting. J Clin Diagn Res.

[4] Gordts B, Vrijens F, Hulstaert F, Devriese S, Van de Sande S (2010). The 2007 Belgian national prevalence survey for hospital-acquired infections. J Hosp Infect.

[5] Sydnor ER, Perl TM (2011). Hospital epidemiology and infection control in acute-care settings. Clin Microbiol Rev.

[6] Loomba PS, Taneja J, Mishra B (2010). Methicillin and Vancomycin Resistant S. aureus in Hospitalized Patients. J Glob Infect Dis.

[7] Centers for Disease Control and Prevention (CDC) (2007). Guidelines for Isolation Precautions: Preventing Transmission of Infectious Agents in Healthcare Settings.

[8] Ducel G, Fabry J, Nicolle L (2002). Prevention of hospital acquired infections. A practical guide.

[9] Patel DA, Patel KB, Bhatt SK, Shah HS (2011). Surveillance of hospital acquired infection in surgical wards in tertiary care centre Ahmedabad, Gujarat. Nat J Commun Med.

[10] Ramasubramanian V, Iyer V, Sewlikar S, Desai A (2014). Epidemiology of healthcare acquired infection – an Indian perspective on surgical site infection and catheter related blood stream infection. Ind J Basic Appl Med Res.

[11] Centers for Disease Control and Prevention (2017). Identifying healthcare-associated infections (HAI) for NHSN surveillance.

[12] Attal R, Deotale V, Potharkar A (2017). Antimicrobial resistance of bacterial isolates from respiratory secretions of ventilator associated pneumonia in the intensive care units of a tertiary care hospital. Int J Curr Res.

[13] Baselski VS, el-Torky M, Coalson JJ, Griffin JP (1992). The standardization of criteria for processing and interpreting laboratory specimens in patients with suspected ventilator-associated pneumonia. Chest.

[14] Mackie TJ, McCartney JE (1996). Practical Medical Microbiology.

[15] Patel JB, Cockerill FR, Alder J, Bradford PA, Eliopoulos GM, Hardy DJ, Hindler JA, Jenkins SG, Lewis JS, Miller LA, Powell M, Swenson JM, Traczewski MM, Turnidge JD, Weinstein MP, Zimmer BL (2014). Performance standards for antimicrobial susceptibility testing; twenty-fourth informational supplement. CLSI document M100-S24.

[16] Shalini S, Kranthi K, Gopalkrishna Bhat K (2010). The microbiological profile of nosocomial infections in the intensive care unit. J Clin Diagn Res.

[17] Singh S, Chaturvedi R, Garg SM, Datta R, Kumar A (2013). Incidence of healthcare associated infection in the surgical ICU of a tertiary care hospital. Med J Armed Forces India.

[18] Moolchandani K, Sastry AS, Deepashree R, Sistla S, Harish BN, Mandal J (2017). Antimicrobial Resistance Surveillance among Intensive Care Units of a Tertiary Care Hospital in Southern India. J Clin Diagn Res.

[19] Anand N, Nagendra Nayak IM, Advaitha MV, Thaikattil NJ, Kantanavar KA, Anand S (2016). Antimicrobial agents' utilization and cost pattern in an Intensive Care Unit of a Teaching Hospital in South India. Indian J Crit Care Med.

[20] Patel MK, Barvaliya MJ, Patel TK, Tripathi C (2013). Drug utilization pattern in critical care unit in a tertiary care teaching hospital in India. Int J Crit Illn Inj Sci.

[21] Mehta Y, Jaggi N, Rosenthal VD, Kavathekar M, Sakle A, Munshi N (2016). Device-associated infection rates in 20 cities of India, data summary for 2004-2013: Findings of the International Nosocomial Infection Control Consortium. Infect Control Hosp Epidemiol. 2016;37:172-81.Mehta Y, Jaggi N, Rosenthal VD, Kavathekar M, Sakle A, Munshi N, Chakravarthy M, Todi SK, Saini N, Rodrigues C, Varma K, Dubey R, Kazi MM, Udwadia FE, Myatra SN, Shah S, Dwivedy A, Karlekar A, Singh S, Sen N, Limaye-Joshi K, Ramachandran B, Sahu S, Pandya N, Mathur P, Sahu S, Singh SP, Bilolikar AK, Kumar S, Mehta P, Padbidri V, Gita N, Patnaik SK, Francis T, Warrier AR, Muralidharan S, Nair PK, Subhedar VR, Gopinath R, Azim A, Sood S. Device-Associated Infection Rates in 20 Cities of India, Data Summary for 2004-2013: Findings of the International Nosocomial Infection Control Consortium. Infect Control Hosp Epidemiol.

[22] Dudeck MA, Weiner LM, Allen-Bridson K, Malpiedi PJ, Peterson KD, Pollock DA, Sievert DM, Edwards JR (2013). National Healthcare Safety Network (NHSN) report, data summary for 2012, Device-associated module. Am J Infect Control.

[23] Deepashree R, Raghavan R, Sastry AS (2017). Implementation of active surveillance system to track hospital-acquired infections in a tertiary care hospital in India. J Curr Res Sci Med.

[24] Wang L, Zhou KH, Chen W, Yu Y, Feng SF (2019). Epidemiology and risk factors for nosocomial infection in the respiratory intensive care unit of a teaching hospital in China: A prospective surveillance during 2013 and 2015. BMC Infect Dis.

[25] Rai V, Rosenthal V, Gan CS, Hasan MS, Lum L, Mansor M, Chuah S, Jamaluddin F, Aziz F, Hoon Y, Zainal Abidin A, Zaman M, Yusoff W, Ponnampala S, Othman A, Zainol H, Zhazali R, Kamarulzaman A (2016). Device-associated infection and mortality rates, bacterial resistance, and length of stay in hospitals of Malaysia: International Nosocomial Infection. Can J Infect Contr.

[26] Masoudifar M, Gouya MM, Pezeshki Z, Eshrati B, Afhami S, Farzami MR, Seifi A (2022). Health care-associated infections, including device-associated infections, and antimicrobial resistance in Iran: The national update for 2018. J Prev Med Hyg.

[27] Mathur P, Tak V, Gunjiyal J, Nair SA, Lalwani S, Kumar S, Gupta B, Sinha S, Gupta A, Gupta D, Misra MC (2015). Device-associated infections at a level-1 trauma centre of a developing nation: impact of automated surveillance, training and feedbacks. Indian J Med Microbiol.

[28] Kołpa M, Wałaszek M, Gniadek A, Wolak Z, Dobroś W (2018). Incidence, microbiological profile and risk factors of healthcare-associated infections in intensive care units: A 10 year observation in a provincial hospital in Southern Poland. Int J Environ Res Public Health.

[29] Khan ID, Basu A, Kiran S, Trivedi S, Pandit P, Chattoraj A (2017). Device-associated healthcare-associated infections (DA-HAI) and the caveat of multiresistance in a multidisciplinary intensive care unit. Med J Armed Forces India.

