# Supplementary material for: Surveillance of health-care associated infections in an intensive care unit at a tertiary care hospital in Central India
Source: GMS Hyg Infect Control. 2023 Nov 29;18:Doc28. doi: 10.3205/dgkh000454 (PMC10726722; doi:10.3205/dgkh000454)
Supplement: Audit form [file HIC-18-28-s-001.pdf]

Attachment 1: Audit form

HOSPITAL ACQUIRED INFECTION SURVEILLANCE FORM (AUDIT)

Patient Name:	H. No.	Age:	Sex:<br>M/ F	ICU/ Ward:
Department:	Admitting Unit:	Dt. Of Adm.		Dt. Of Adm. To ICU -
Provisional Diagnosis:		Final Diagnosis:		
Outcome:	Transfer out to ward/unit name & date	LAMA on:	Discharged on	Expired on:

Risk factor/C0-morbidities: (Circle features present at admission)

DM	HTN	CLD	CKD	HIV	TB	Transplantation	Immunosuppressant	any other

Type of Surgery- **Date of Surgery:**

Type of device used and Device Days

Intervention	Date of Insertion	Date of Removal	Re-insertion	Removal
Urinary Catheter				
Mechanical Ventilation/ET tube				
Tracheostomy				
CVC- Jugular/ Subclavian/Femoral/PICC				
Surgical Site Drainage tube				
Dialysis Sheath				

Daily Monitoring

		HD-1	D-2	D-3	D-4	D-5	D-6	D-7	D-8	D-9	D-10	D-11	D-12	D-13	D-14	D-15
**HAI**	**Date**															
	Temperature															
**CA-UTI**	Catheter present															
	Suprapubic Tenderness															
	Loin pain															
	*1.Urgency, 2.Frequency 3. Dysuria															
**CLA BSI**	CL (central line) present															
	Chills															
	Hypotension (SBP ≤ 90)															
**VAE**	MV (mechanical ventilator) present															
	$PEEP_{dm}$															
	$FiO2_{dm}$															
	WBC count															
	New antibiotics															
**SSI**	Purulent discharge at site															
	Clinician's diagnosis															
	Tenderness, swelling, erthema, heat															
	**Abscess at site															

- *To be reported only when urinary catheter is not in place
- **Detected by physical exam/histopathological exam/imagingdm-daily minimum

Attachment to: Lohiya R, Deotale V. Surveillance of health-care associated infections in an intensive care unit at a tertiary care hospital in Central India. GMS Hyg Infect Control. 2023;18:Doc28. DOI: 10.3205/dgkh000454

Microbiology Culture Report (Site-specific culture and blood culture; to be filled even culture is negative)

Date of Sample collection	Sample	Organism isolated	Colony count	AST report

(S- sensitive, R- resistant, Ak- Amikacin, G- Gentamicin, CFS- Cefoperazone-sulbactum, Ci-Ceftriaxone, Ca-Ceftazidime, Cx-Cefoxitin, Ox-Oxacillin, M-Meropenem, PIT-Piperacillin-tazobactum, Cf-Ciprofloxacin, N-Nitrofurantoin, E-erythromycin, P-Penicillin, T-tetracycline)

BUNDLE CARE AUDIT																
		D1	D2	D3	D4	D5	D6	D7	D8	D9	D10	D11	D12	D13	D14	D15
**Urinary catheter care bundle**																
Closed drainage system																
Urinary catheter secured																
Drainage bag above floor & Below bladder level																
Catheter care (aseptic)	hand hygiene															
	Vaginal/meatal care															
	perineal care															
Single use glove while handling/emptying (No contact b/t jug and bag)																
Separate jug for collecting																
Assessment of readiness to remove – documented?																
**Central line bundle**																
Daily aseptic CL care during handling	Hand hygiene															
	Alcohol hub decontamination															
	CHG 2% for Dressing changes															
Any local signs of infection?																
Dressing changed?																
Assessment of readiness to remove – documented?																
**Ventilator bundle**																
Head and elevation 30^0																
Adherence to hand hygiene																
Daily oral care (CHG 2%)																
Need of PUD prophylaxis assessed?																
DVT prophylaxis																
Assessment of readiness to remove – documented?																

ICN Name and Signature with date

HOSPITAL ACQUIRED INFECTION SURVEILLANCE FORM (ADULT) Page-2

CAUTI(CATHETER ASSOCIATED UTI) **Date of Event (DOE)-**

1.Urinary Catheter Criteria	Patient has indwelling urinary catheter in place for >2 calendar day						Yes/No
	Or if removed: Urinary catheter was in place on the day of sample collection or the day before						Yes/No
2. Symptom Criteria	At least one of the following						
	Fever (>100.4°F) ☐	Suprapubic tenderness ☐	Loin Pain ☐	Urgency ☐	Frequency ☐	Dysuria ☐	Yes/No
3. Urine culture Criteria	Positive urine culture<br>(Not more than two organisms with at least one organism having ≥ 10^5 CFU/ml)						Yes/No
4. Blood culture criteria	No symptoms<br>Positive blood culture (with one matching organism to urine culture)						Yes/No
**Final diagnosis**	Symptomatic CAUTI (criteria-1 + 2 + 3) ☐			ABUTI (Asymptomatic bacteremic UTI) (criteria- 1+4) ☐			

CLABSI (CENTRAL LINE ASSOCIATED BLOODSTREAM INFECTION) **Date of Event (DOE)-**

1. Central line criteria	Patient has central line in place for 2 days or more					Yes/No
	Or If removed: Central line was in place on the day of sample collection or the day before					Yes/No
2.Pathogen	Pathogen identified from one blood culture (Not related to infection at any other site)					Yes/No
3.Commensal (culture+ve & symptoms)	Commensal grown from two blood cultures (Not related to infection at other sites) and symptoms					Yes/No
	3a- (Adult) At least one:	Fever (>100.4°F) ☐	Chills ☐	Hypotension (SBP ≤ 90) ☐		Yes/No
	3b- (<1 year) At least one:	Fever (>100.4°F) ☐	Hypothermia ☐	Apnea ☐	Brady-cardia ☐	Yes/No
**Final diagnosis**		LCBI-2 (1+3a) ☐	LCBI-2 (1+3a) ☐	LCBI-3 (1+3b) ☐	Date of onset	

VAE (VENTILATOR ASSOCIATED EVENT): **Date of Event (DOE)-**

**MV criteria**	Patient has mechanical ventilator (MV) in place for 2 days or more			Yes/No
	Or If removed: MV was in place on the day of sample collection or the day before			Yes/No
**Baseline**	Patient has a baseline period of stability or improvement on the ventilator, defined by ≥ 2 days of Stable or decreasing daily minimum PEEP (5 or less) or FiO_2 (40% or less)			Yes/No
**VAC**	Increase in FiO_2dm by ≥ 20 % for ≥ 2 days ☐			Yes/No
	**Or** Increase in PEEPdm by ≥ 3 cm of H_2O for ≥ 2 days ☐			
**i-VAC**	Temperature >100.4°F or < 96.8 °F, OR WBC ≥ 12, 000 cells/mm or ≤ 4, 000 cells/mm ☐			Yes/No
	**And** A new antimicrobial agent is started within 5 days of DOE, and is continued for ≥ 4 days ☐			
**P-VAP**	Culture positive with significant growth<br>(ET aspire- ≥ 10^5 CFU/ml), (BAL, lung tissue - ≥ 10^4) (brush - ≥ 10^3) ☐			Yes/No
	Direct smear-Purulent resp. secretions (PC>25/LPF, EC<10/LFP) **AND** Culture positive (any growth)<br>(from sputum, ET aspirate, BAL, lung tissue or brush) ☐			
**Final diagnosis**	**VAC** (Ventilator associated condition) ☐	**i- VAC** (infection related ventilator associated complication) ☐	**P- VAP** (Possible ventilator Associated pneumonia) ☐	

SSI (SURGICAL SITE INFECTION): **Date of Event (DOE)-**

1.	Patient had a surgery within past 30 days or<br>Surgery within 90 days if implant in place or breast, cardiac surgery of herniorrhaphy								Yes/No
2.	Wound class (Tick appropriate)		Clean ☐	Clean contaminated ☐		Contaminated ☐		Dirt ☐	
3.	PATOS (Present At the Time of Surgery)- visible pus/abscess at operation site; documented in OT note								Yes/No
4.	Any one of the following								
	SI-SSI (Superficial Incisional)	Any one of the following:<br>1. Purulent drainage from superficial incision ☐<br>2. Positive culture (pus/tissue) ☐<br>3. Incision opened, culture not sent but patient has at least one symptoms: pain or Tenderness; localized swelling; erythema; or heat. ☐<br>4. Clinician's diagnosis as S-SSI ☐							Yes/No
	DI- SSI (Deep Incisional)	Any one of the following:<br>1. Purulent drainage from deep incision ☐<br>2. Positive culture (pus/tissue) ☐<br>3. Incision dehisces spontaneously or opened deliberately, culture not sent but patient has at least one symptoms: fever (>100.4°F), pain or tenderness. ☐<br>4. Abscess involving the deeper incision found at physical exam/histopath/imaging ☐							Yes/No
	Organ/space SSI	Any of the following:<br>1. Purulent drainage from drain through the organ or space ☐<br>2. Positive culture (pus/tissue) from the discharge from drain/organ or space ☐<br>3. Abscess involving the organ or space found at physical exam/histopath/imaging ☐							Yes/No

Attachment to: Lohiya R, Deotale V. Surveillance of health-care associated infections in an intensive care unit at a tertiary care hospital in Central India. GMS Hyg Infect Control. 2023;18:Doc28. DOI: 10.3205/dgkh000454
