# Supplementary material for: Surveillance of health-care associated infections in an intensive care unit at a tertiary care hospital in Central India
Source: GMS Hyg Infect Control. 2023 Nov 29;18:Doc28. doi: 10.3205/dgkh000454 (PMC10726722; doi:10.3205/dgkh000454)
Supplement: Daily Round Checklist (ICN) [file HIC-18-28-s-002.pdf]

Attachment 2: Daily Round Checklist (ICN)

**DAILY ICN ROUND FORMAT – ICU –<br>CHECKLIST FOR HOSPITAL INFECTION CONTROL ROUNDS, KHS Sevagram**																
Date	Total Patient Days	ON VENTI.	ON CVC	ON CATHE.	Fever cases	Hand Hygiene	PPE	BMW	**Disinfectantsolu.**	**Cleanliness**	**Spill Kit/ first aid box**	**Dressing Trolly**	**Crash cart**	**NSI**	**Sign. Of I/C**	**Sign. Of Microbiologist**

Attachment to: Lohiya R, Deotale V. Surveillance of health-care associated infections in an intensive care unit at a tertiary care hospital in Central India. GMS Hyg Infect Control. 2023;18:Doc28.
DOI: 10.3205/dgkh000454
