# Supplementary material for: Surveillance of health-care associated infections in an intensive care unit at a tertiary care hospital in Central India
Source: GMS Hyg Infect Control. 2023 Nov 29;18:Doc28. doi: 10.3205/dgkh000454 (PMC10726722; doi:10.3205/dgkh000454)
Supplement: ICU Checklist [file HIC-18-28-s-003.pdf]

Attachment 3: ICU Checklist
Intensive Care Unit

Name of Unit: Date & Time visited:

Does the civil construction meet basic criteria for ICU?	Yes	No
Are the internal setting (eg. Distance between beds, oxygen supply etc.) and human resource (eg. Doctor:patient ratio, nurse:patient ratio etc.) adequate?	Yes	No
Is AC functioning?	Yes	No
If not, what is the alternative method for air circulation?	Yes	No
Is there a written SOP for ICU?	Yes	No
Are all staff and doctors trained in Hospital Infection control?	Yes	No
Are all staff members are aware of PPE?	Yes	No
Are swabs sent on routine basis to the Microbiology Dept. as a part of Active Hospital Infection Surveillance?	Yes	No
If yes, how frequently?		
Are all staff members aware of Hospital infection Committee?	Yes	No
Is there any refusal to cooperate with ICN at her routine visit?	Yes	No
What is the frequency of sweeping the floor?		
What is the frequency of changing linen?		
Are visiting hours being maintained?	Yes	No
What is the timing of visiting hours?		
Are invasive procedures done during visiting hours?	Yes	No
Are shoes available for visitors?	Yes	No
Are aprons available for visitors?	Yes	No
Is a hospital infection register available?	Yes	No
Is a CAUTI register available?	Yes	No
Is an invasive procedure register available?	Yes	No
Are gloves worn:		
Whenever in contact with blood and body fluids?	Yes	No
When specimens, soiled linen, body fluids, secretion as well as surfaces, materials or objects exposed to them have to be handled?	Yes	No
Whenever the skin is not intact?	Yes	No
Double gloves are recommended for high risk patients?	Yes	No
Are gloves worn during:		
Venepuncture, Annulation, Urinary catheterization, Nursing of immunocompromised patients, Suctioning (tracheal), Blood sampling, Vaginal delivery, Dental procedures?	Yes	No
What material is used for routine hand wash in the ward?		
Are injection syringe needles recapped?	Yes	No
How frequently are IV sets replaced?		
How frequently is tubing used to administer blood/body products, fat emulsion replaced?		
How frequently are catheters replaced routinely?		
Are catheters flushed on blockade?	Yes	No
Are Ambubags cleaned with detergent and water, dried and sterilized before reuse?	Yes	No
Are Arterial catheters reused?	Yes	No
Are baby bottles and teats returned to CSSD or washed in hot detergent and water, rinsed and immersed in Milton fluid, freshly made up from tablets according to manufacturer's instructions?	Yes	No
Are bedpans and urinals cleaned and disinfected with 0.5% sodium hypochlorite or hot water and air dried before reuse?	Yes	No
Are cradles cleaned with detergent and water and dried?	Yes	No
Are reusable drainage bottles used?	Yes	No

Attachment to: Lohiya R, Deotale V. Surveillance of health-care associated infections in an intensive care unit at a tertiary care hospital in Central India. GMS Hyg Infect Control. 2023;18:Doc28. DOI: 10.3205/dgkh000454

If yes, are they rinsed and returned to CSSD before reuse?	Yes	No
Are Ear Pieces for autoscore, Earphones cleaned with detergent and water and dried before reuse?	Yes	No
Are leads and monitors dismantled to smallest components and cleaned with detergent and water and dried?	Yes	No
Are sterilized instruments used?	Yes	No
Are instruments returned to CSSD after single use?	Yes	No
Are sphygmomanometer cuff after use in isolation, laundered in washing machine?	Yes	No
Are sputum pots disposable with close-fitting lid and discarded into clinical waste for incineration?	Yes	No
Are suction bottles sealed when 75% full and placed in yellow plastic bag?	Yes	No
If re-usable, are they cleaned with sodium hypochlorite, dried and changed daily, as well as in between each patient?	Yes	No
Are dressing trolleys cleaned daily with detergent and water?	Yes	No
After each use are they wiped with 70% isopropyl alcohol?	Yes	No
Are color-coded buckets available and being promptly used?	Yes	No
Are color-coded posters displayed along with them?	Yes	No
Are all staff members on duty aware of color coding?	Yes	No
Are buckets being emptied every day?	Yes	No
Are needle destroyers available and functioning?	Yes	No

Staff Nurse I/C

HIC Personne

Attachment to: Lohiya R, Deotale V. Surveillance of health-care associated infections in an intensive care unit at a tertiary care hospital in Central India. GMS Hyg Infect Control. 2023;18:Doc28. DOI: 10.3205/dgkh000454
