# Supplementary material for: Surveillance of health-care associated infections in an intensive care unit at a tertiary care hospital in Central India
Source: GMS Hyg Infect Control. 2023 Nov 29;18:Doc28. doi: 10.3205/dgkh000454 (PMC10726722; doi:10.3205/dgkh000454)
Supplement: Checklist for Urinary Catheter [file HIC-18-28-s-004.pdf]

Attachment 4: Checklist for Urinary Catheter

Section A. General information		
Patient ID	Patient Name	
Facility Name	Surveillance unit	Date of admission to surveillance unit (dd/mm/yyyy)

Section B. Daily check

Date (dd/mm/yyyy)	Catheter Day	Was the Catheter reviewed for necessity today?	Signature of the shift nurse	Was the Catheter checked for kinking, leakage and position of urine bag?	Catheter care given	Signature of day shift nurse	During the day shift?	Signature of day shift nurse	During the night shift?	Signature of night shift nurse
		□ Yes □ No		□ Yes □ No	□ Yes □ No		□ Yes □ No		□ Yes □ No	
		□ Yes □ No		□ Yes □ No	□ Yes □ No		□ Yes □ No		□ Yes □ No	
		□ Yes □ No		□ Yes □ No	□ Yes □ No		□ Yes □ No		□ Yes □ No	
		□ Yes □ No		□ Yes □ No	□ Yes □ No		□ Yes □ No		□ Yes □ No	
		□ Yes □ No		□ Yes □ No	□ Yes □ No		□ Yes □ No		□ Yes □ No	
		□ Yes □ No		□ Yes □ No	□ Yes □ No		□ Yes □ No		□ Yes □ No	
		□ Yes □ No		□ Yes □ No	□ Yes □ No		□ Yes □ No		□ Yes □ No	
		□ Yes □ No		□ Yes □ No	□ Yes □ No		□ Yes □ No		□ Yes □ No	
		□ Yes □ No		□ Yes □ No	□ Yes □ No		□ Yes □ No		□ Yes □ No	
		□ Yes □ No		□ Yes □ No	□ Yes □ No		□ Yes □ No		□ Yes □ No	
		□ Yes □ No		□ Yes □ No	□ Yes □ No		□ Yes □ No		□ Yes □ No	
		□ Yes □ No		□ Yes □ No	□ Yes □ No		□ Yes □ No		□ Yes □ No	
		□ Yes □ No		□ Yes □ No	□ Yes □ No		□ Yes □ No		□ Yes □ No	
		□ Yes □ No		□ Yes □ No	□ Yes □ No		□ Yes □ No		□ Yes □ No	
		□ Yes □ No		□ Yes □ No	□ Yes □ No		□ Yes □ No		□ Yes □ No	

Attachment to: Lohiya R, Deotale V. Surveillance of health-care associated infections in an intensive care unit at a tertiary care hospital in Central India. GMS Hyg Infect Control. 2023;18:Doc28.
DOI: 10.3205/dgkh000454
