# Supplementary material for: Surveillance of health-care associated infections in an intensive care unit at a tertiary care hospital in Central India
Source: GMS Hyg Infect Control. 2023 Nov 29;18:Doc28. doi: 10.3205/dgkh000454 (PMC10726722; doi:10.3205/dgkh000454)
Supplement: Checklist for Central Line [file HIC-18-28-s-005.pdf]

Attachment 5: Checklist for Central Line

**Section A. General information**									
Patient ID				Patient Name					
Facility Name		Surveillance unit			Date of admission to surveillance unit (dd/mm/yyyy)				
Date (dd/mm/yyyy)	Central line Day	Was the Central line reviewed for necessity today	Signature of the shift nurse	Was the dressing checked for soiling, dampening, and loosening today?	Signature of the shift nurse	Was the access port scrubbed with an antiseptic each time before use?			
						During the day shift?	Signature of the shift nurse	During the night shift?	Signature of night-shift nurse
		□ Yes □ No		□ Yes □ No		□ Yes □ No		□ Yes □ No	
		□ Yes □ No		□ Yes □ No		□ Yes □ No		□ Yes □ No	
		□ Yes □ No		□ Yes □ No		□ Yes □ No		□ Yes □ No	
		□ Yes □ No		□ Yes □ No		□ Yes □ No		□ Yes □ No	
		□ Yes □ No		□ Yes □ No		□ Yes □ No		□ Yes □ No	
		□ Yes □ No		□ Yes □ No		□ Yes □ No		□ Yes □ No	
		□ Yes □ No		□ Yes □ No		□ Yes □ No		□ Yes □ No	
		□ Yes □ No		□ Yes □ No		□ Yes □ No		□ Yes □ No	
		□ Yes □ No		□ Yes □ No		□ Yes □ No		□ Yes □ No	
		□ Yes □ No		□ Yes □ No		□ Yes □ No		□ Yes □ No	
		□ Yes □ No		□ Yes □ No		□ Yes □ No		□ Yes □ No	
		□ Yes □ No		□ Yes □ No		□ Yes □ No		□ Yes □ No	
		□ Yes □ No		□ Yes □ No		□ Yes □ No		□ Yes □ No	
		□ Yes □ No		□ Yes □ No		□ Yes □ No		□ Yes □ No	
		□ Yes □ No		□ Yes □ No		□ Yes □ No		□ Yes □ No	

Attachment to: Lohiya R, Deotale V. Surveillance of health-care associated infections in an intensive care unit at a tertiary care hospital in Central India. GMS Hyg Infect Control. 2023;18:Doc28.
DOI: 10.3205/dgkh000454
